# Donkey Milk Fermentation by *Lactococcus lactis* subsp. *cremoris* and *Lactobacillus rhamnosus* Affects the Antiviral and Antibacterial Milk Properties

**DOI:** 10.3390/molecules26165100

**Published:** 2021-08-23

**Authors:** Simona Cirrincione, Anna Luganini, Cristina Lamberti, Marcello Manfredi, Laura Cavallarin, Maria Gabriella Giuffrida, Enrica Pessione

**Affiliations:** 1 Institute of the Science of Food Production—National Research Council, Largo Braccini 2, 10095 Grugliasco, Torino, Italy; simona.cirrincione@ispa.cnr.it (S.C.); laura.cavallarin@ispa.cnr.it (L.C.); gabriella.giuffrida@ispa.cnr.it (M.G.G.); 2 Department of Life Science and System Biology, University of Torino, Via Accademia Albertina 13, 10123 Torino, Italy; anna.luganini@unito.it (A.L.); enrica.pessione@unito.it (E.P.); 3 Center for Translational Research on Autoimmune and Allergic Disease—CAAD, University of Piemonte Orientale, Corso Trieste 15/A, 28100 Novara, Italy; marcello.manfredi@uniupo.it

**Keywords:** antimicrobial activity, bioactive peptides, nutraceuticals, herpes simplex virus, DPPH

## Abstract

Background: Milk is considered an important source of bioactive peptides, which can be produced by endogenous or starter bacteria, such as lactic acid bacteria, that are considered effective and safe producers of food-grade bioactive peptides. Among the various types of milk, donkey milk has been gaining more and more attention for its nutraceutical properties. Methods: *Lactobacillus rhamnosus* 17D10 and *Lactococcus lactis* subsp. *cremoris* 40FEL3 were selected for their ability to produce peptides from donkey milk. The endogenous peptides and those obtained after bacterial fermentation were assayed for their antioxidant, antibacterial, and antiviral activities. The peptide mixtures were characterized by means of LC-MS/MS and then analyzed in silico using the Milk Bioactive Peptide DataBase. Results: The peptides produced by the two selected bacteria enhanced the antioxidant activity and reduced *E. coli* growth. Only the peptides produced by *L. rhamnosus* 17D10 were able to reduce *S. aureus* growth. All the peptide mixtures were able to inhibit the replication of HSV-1 by more than 50%. Seventeen peptides were found to have 60% sequence similarity with already known bioactive peptides. Conclusions: A lactic acid bacterium fermentation process is able to enhance the value of donkey milk through bioactivities that are important for human health.

## 1. Introduction

Food proteins, although physiologically essential *per se*, can display bioactive properties when their peptides are decrypted by enzymatic hydrolysis, which can be performed by both gastrointestinal and microbial enzymes [1,2].

Among all the different food products, milk is considered the most important source of bioactive peptides (BP) [3] due to its high protein content. BP from dairy products can originate in two different phases: (i) during milk fermentation and cheese ripening, from endogenous or starter bacteria, or (ii) in the human intestinal tract, as the result of the action of both digestive enzymes and endogenous microbiota [4]. Different microbial genera, including yeasts and bacteria, are able to decrypt peptides from milk proteins. Among bacteria, lactic acid bacteria (LAB) have been reported as an efficient and safe medium to produce food-grade hydrolytic enzymes and BP [5,6]. The quantity and the quality of the decrypted peptides depend to a great extent on the specific proteolytic pathways used by the added LAB. Lactobacilli and lactococci are both particularly suitable for milk fermentation, since they possess efficient proteolytic systems [7] that are highly effective for caseins and whey proteins hydrolysis [8].

Furthermore, LAB often provide additional health-promoting effects, due to their probiotic activities concerning SCFA (short-chain fatty acid) and GABA (γ-aminobutyric acid) production, selenium fixing, and EPS synthesis and secretion [9]. As far as milk caseins are concerned, *L. helveticus*, *L. rhamnosus*, *L. bulgaricus*, and *L. lactis* subsp. *cremoris* strains seem to be the most suitable for BP production [10]. However, most LAB strains can also support the hydrolytic cleavage of milk whey proteins (α-lactalbumin, β-lactoglobulin, lactoferrin, and immunoglobulins), thus generating BP [11,12], such as hypocholesterolaemic, antihypertensive, and opioid peptides [13], as well as antioxidant and immune-modulating ones [14,15]. In addition, many food-derived bioactive peptides have recently also shown anticancer and antidiabetic properties that are useful to control widespread pathologies that undermine human health [16].

Most of the studied BP are derived from cow milk or from the related dairy products, since it accounts for more than 80% of the world’s milk production. However, milk from other ruminant and non-ruminant mammals, which are often consumed in specific geographical areas, have been considered for their particular nutritional properties. For example, the milk from sheep and buffalo constitutes a good raw material for cheese making, due to its high casein and fat contents [17]. Conversely, the milk from donkeys and horses (which has the most comparable protein and fat composition with human milk) is characterized by low casein and fat contents [17], and for this reason, it is not suitable for cheese making. Donkey milk (DM) is used successfully as an alternative food for infants who suffer from cow milk protein allergy (CMPA), which is a common food allergy in childhood that shows a prevalence of up to 3% during the first year of life [18]. Moreover, a number of therapeutic properties (suitable for treating metabolic, gastrointestinal, and liver problems, and for treating or preventing atherosclerosis, arthritis, and cancer) have been attributed to DM [19].

In the last few years, interest in DM as a nutraceutical food has also led to the characterization of the DM peptidome and its bioactivities. Tidona et al. [20] described the DM peptidome obtained after the digestion with human gastrointestinal enzymes, while Piovesana et al. [21] investigated the peptides that occur naturally in DM and identified more than one thousand peptides by means of LC-MS/MS, some of which have antioxidant and ACE-inhibitory properties. However, to the best of our knowledge, only a few researchers have reported on the characterization of the peptides released by the bacterial proteolytic system and their bioactivities in fermented DM [22].

In this scenario, given the interest in the discovery of BP and the central role of milk in such a context, the proteolytic system of selected LAB was explored to find peptides from donkey milk proteins that displayed antibacterial, antiviral, and antioxidant properties. Furthermore, high-performance liquid chromatography, coupled with tandem mass spectrometry (LC–MS/MS) detection, was used to investigate the peptide sequences obtained after the protein digestion of donkey milk.

## 2. Results and Discussion

### 2.1. Microbial Characterization of the Donkey Milk

Five different cultivable bacteria were isolated from DM: *Pseudomonas fluorescens*, *Lactococcus lactis*, *Lelliotta amingenes*, *Lysinibacillus sphericus*, and *Serratia* sp.

*P. fluorescens* was identified, by means of biochemical and metabolic testing (API 20E), with a reliable 99.9% result. This bacterium is among the most important psychrotrophic Gram-negative bacteria responsible for undesirable flavors in cow milk and dairy products [23]. It is well known that the refrigeration of raw milk for longer periods than 48 h causes an increase in the psychrotrophic population [24]. Moreover, the lipolytic and proteolytic enzymes synthesized by *P. fluorescens* are not inactivated by heat treatments, thus causing rancidity in pasteurized and UHT milk, milk powder, and cheese [25]. *Lactococcus lactis*, *Lelliotta amnigena*, *Lysinibacillus Sphaericus,* and *Serratia* sp. were identified by means of 16s rRNA analyses. The isolation of *Lactococcus lactis* from DM constitutes an interesting starting point for further applications, considering that Gram-positive LAB species are often active components of the microflora of cow milk that influence nutritional aspects and also act as biocontrol agents [26]. *Lelliottia amnigena* is a Gram-negative strain that is originally a member of the Enterobacter genus. Lelliottia species are often isolated from water, from elm trees exhibiting “wetwood” disease symptoms and, albeit rarely, from clinical samples [27]. Only a few studies regarding the isolation of this specific bacterium in milk or dairy products are present in the literature [28], thus indicating that the presence of *Lelliottia amnigena* in DM is probably the result of water contamination. *Lysinibacillus sphaericus*, which is generally known as *Bacillus sphaericus,* is a Gram-positive, mesophilic, rod-shaped bacterium. Under harsh conditions, it can form dormant endospores that have a much longer “life-span” and higher resistance against chemical and mechanical stress than vegetative cells [29]. This bacterium, together with other aerobic spore-forming bacteria, was isolated and characterized from raw cow milk, and its ability to reduce nitrate and its poor proteolytic activity have been demonstrated [24]. The *Serratia* strain (the use of a universal primer did not allow it to be identified at the species level [30]) is a Gram-negative, psychrotrophic bacterium, which is often isolated as a milk contaminant in goat and sheep milk [31]. Similar to *P. fluorescens*, the Serratia genus is responsible for the spoilage of milk as a result of lipolytic activity [32]. Moreover, different Serratia species were found to be involved in animal mastitis [33], and their presence is therefore probably due to milk contamination during milking.

The high variability, in terms of genera and species isolated from DM, indicates that similar to other kinds of mammalian milk, there are multiple sources of contamination along the dairy chain: water, soil, air, feces, udder, milking equipment, etc. Therefore, more controls are required in the milking, transport, and storage phases as well as efficient sterilization methods in order to reduce the bacteria involved in milk spoilage and/or human disorders.

### 2.2. Screening of the LAB for Peptide Production

With the aim of using the LAB for DM fermentation and their proteolytic enzymes to produce bioactive peptides, we drastically reduced, or even eliminated, the endogenous microflora of the starting milk. For this reason, a pasteurization process, based on two heat treatment steps, was applied to obtain sterilized milk samples before LAB inoculation (see the M&M section). Five food-isolated LAB strains (*Lactobacillus reuteri* Lb2 BM-DSM, *Lactobacillus helveticus* 6E8, *Lactobacillus rhamnosus* 17D10, *Lactococcus lactis* subsp. *cremoris* 40FEL3, *Lactococcus lactis* subsp. *lactis* MG1363) belonging to our culture collection were evaluated for their ability to grow in DM and to produce peptides. Since milk turbidity interferes with the measurement of OD_595_, bacterial growth was assessed in the DM after 24 h by considering the lowering of the pH in the medium and the L-lactic acid production. Moreover, an OPA quantification assay was performed to determine the content of the peptides released in the medium by each selected strain. It is worth recalling that the peptide content reflects the presence of both peptides that occur naturally as a result of spontaneous milk protein degradation and those generated by the bacterial proteolytic system. As reported in Table 1, the pH value reached at the end of the fermentative process was lower for *L. rhamnosus* 17D10 and *L. lactis* subsp. *cremoris* 40FEL3 than for the other tested LAB. Moreover, these bacteria were responsible for a higher production of L-lactic acid and peptides than the other strains, thus indicating a good growth in DM. All the evaluated parameters indicate that these two strains were well-adapted to the DM and show an active proteolytic system that generated a two-fold higher number of peptides than the other tested strains. Considering the obtained results, *L. rhamnosus* 17D10 and *L. lactis* subsp. *cremoris* 40FEL3 were selected for peptide production. The purified peptide mixtures, obtained from the DM (CtlP) and DM fermented with *L. rhamnosus* 17D10 (LrP) and *L. lactis* subsp. *cremoris* 40FEL3 (LcP), were analyzed to assess the antioxidant, metal-chelating, antibacterial, and antiviral activities.

### 2.3. Antioxidant and Metal-Chelating Activity

**Figure 1:**
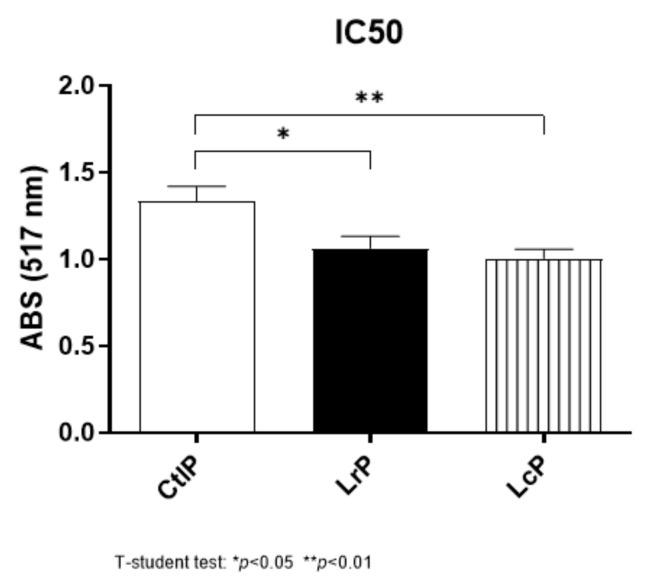
Antioxidant activity of the peptides from donkey milk inoculated with *L. rhamnosus* 17D10 (LrP), *L. lactis* subsp. *cremoris* 40FEL3 (LcP), and not inoculated (CtlP). The antioxidant activity is expressed as the half-maximal inhibitory concentration (IC50).

The antioxidant properties of the peptide mixture, evaluated as DPPH scavenging activity, highlighted that the milk fermentation by LrP and LcP resulted in a 25% enhancement of the antioxidant activity, compared to the non-fermented DM (CtlP) (Figure 1). These results show the presence of an intrinsic antioxidant activity of endogenous DM peptides, regardless of the peptides released from LAB fermentation, as previously reported by Zenezin Chiozzi et al. [34]. The ability of LAB to improve the antioxidant properties after fermentation has been widely proved not only in milk [35] but also in other food products, including quinoa and broccoli [36,37].

As far as the metal-chelating properties are concerned, no activity was observed for either copper or iron, in both the fermented and non-fermented DM.

### 2.4. Antibacterial Activity

The antibacterial activity of the peptide mixtures obtained after DM fermentation was evaluated against two pathogenic strains of *Escherichia coli* and *Staphylococcus aureus*, with the aim of highlighting any possible differences in the peptide effectiveness, according to the membrane/cell wall structure of both Gram-negative and Gram-positive bacteria.

**Figure 2:**
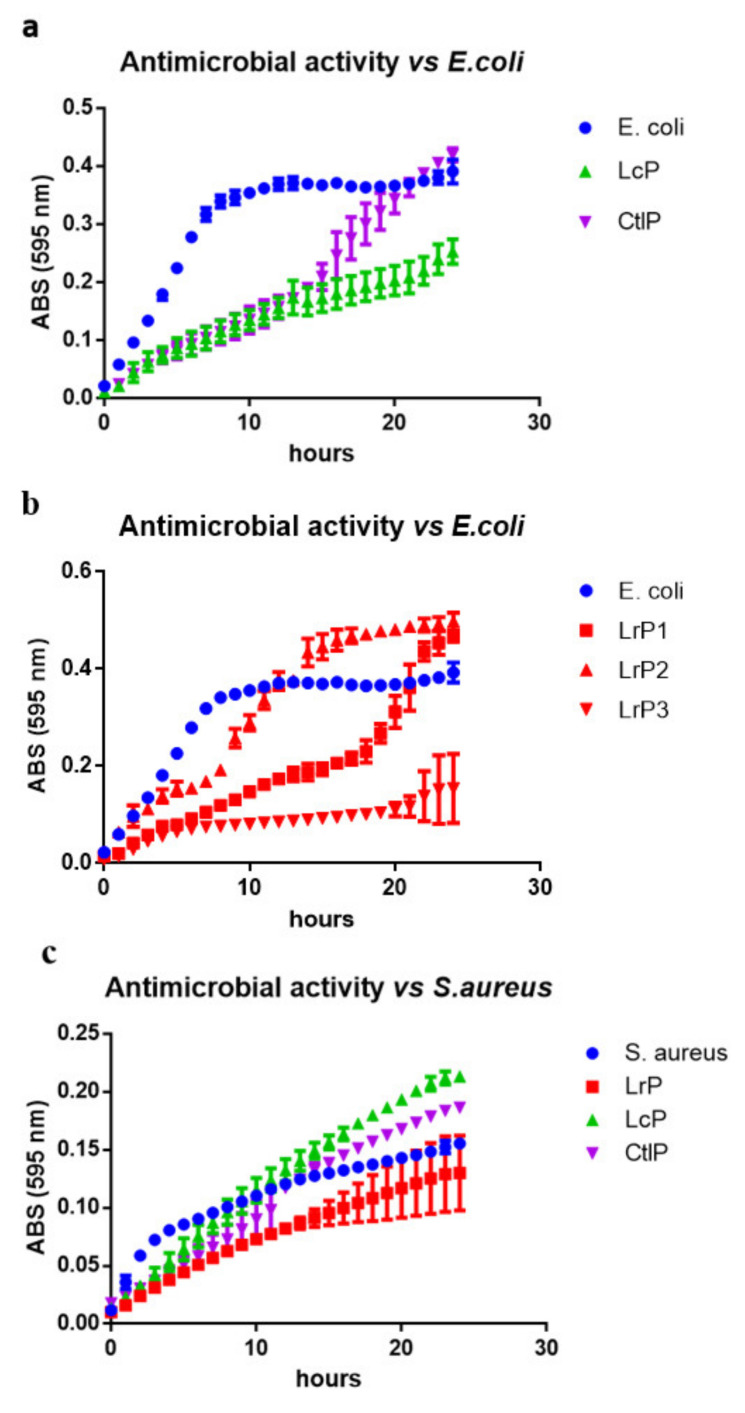
Antibacterial activity of the peptides from donkey milk inoculated with *L. rhamnosus* 17D10 (LrP), *L. lactis* subsp. *cremoris* 40FEL3 (LcP), and not inoculated (CtlP). The antibacterial activity was evaluated against *E. coli* and *S. aureus* clinical isolates as means of pathogen growth inhibition. (**a**) Antimicrobial activity of LcP and CtlP samples vs. *E. coli*; (**b**) the three biological replicates of LrP samples vs. *E. coli* were reported individually due to their high variability; (**c**) antimicrobial activity of LrP, LcP, and CtlP samples vs. *S. aureus*.

The antimicrobial activity was evaluated as a variation of the bacterial growth pattern in the presence of the peptide mixtures (LrP, LcP, and CtlP), compared with the reference growth in the absence of peptides. The growth curves displayed in (Figure 2a–c) show that the LrP and LcP were able to exert an antibacterial effect on both *E. coli* and *S. aureus* cells. An extremely variable behavior was in particular observed for LrP activity vs. *E. coli*, for which the three biological replicates are depicted individually (Figure 2b).

This result is not surprising considering that the fermentation process, even though conducted under standardized conditions, is affected by the variability of the DM composition and by the complexity of the proteolytic system of the LAB. This evidence is also supported by the LC-MS/MS analysis, which highlighted the production of peptides with different sequences among the replicates (see the next paragraph).

The bacterial growth of *E. coli* proved to be affected to a great extent by both the LcP and CtlP samples during the logarithmic phase (Figure 2a). The *E. coli* growth rate (µ) observed in the reference curve is in fact 1.55-fold and 1.40-fold higher than LcP and CtlP during the exponential phase, respectively. However, while the LcP extract exerted a long-lasting effect, a restart of the growth of *E. coli* was observed earlier for CtlP than LcP, and the same amount of biomass was reached as for the reference curve. Different scenarios were observed for *S. aureus* (Figure 2c). LrP was able to reduce *S. aureus* growth longer than LcP or CtlP, both of which showed a transient growth-limiting effect, which only lasted a few hours. Paradoxically, the effect of LcP and CtlP on *S. aureus* growth resulted in a higher final biomass than that of the reference growth curve.

These results highlight that the released peptides exerted a transient bacteriostatic effect on the growth of both pathogens. According to the literature, these peptides could act by interfering with the membrane transporters, thus leading to an altered accumulation of nutrients (e.g., acids, amino acids, and glycerol) during the Lag phase. As already demonstrated, antimicrobial peptides (AMPs) mainly exert their effect by interacting with the cell envelope, the cytoplasmic or outer membrane, or with the peptidoglycan cell-wall [38]. AMPs are generally amphipathic molecules, with a hydrophobic moiety and a positively charged domain that first bind to teichoic acids in Gram-positive bacteria or to LPS in Gram-negative bacteria [39] and then interact with negatively charged bacterial membranes.

Our experiments, apart from confirming the nutraceutical properties of DM, provided the first evidence of the antimicrobial activity of fermented DM with *L. rhamnosus* and *L. lactis* subsp. *cremoris*. These results may be considered promising in view of developing fermented drinks based on donkey milk with enriched antimicrobial properties.

### 2.5. Antiviral Activity

**Figure 3:**
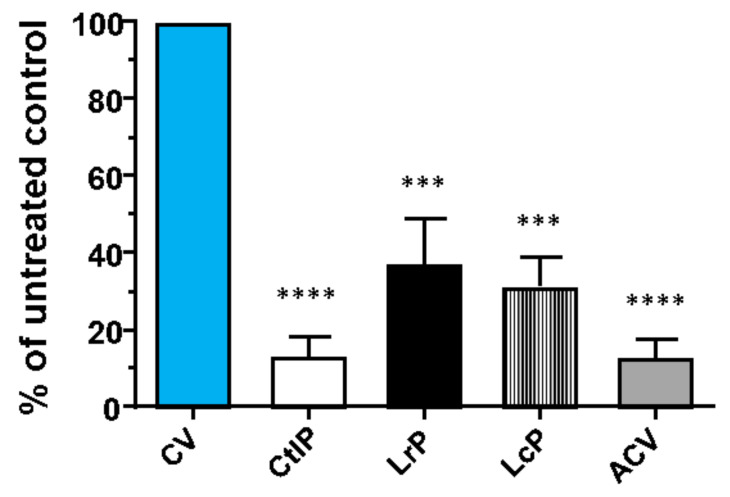
Antiviral activity of the peptides from donkey milk inoculated with *L. rhamnosus* 17D10 (LrP), *L. lactis* subsp. *cremoris* 40FEL3 (LcP), and not inoculated (CtlP). The antiviral activity was evaluated against an HSV-1 clinical isolate. As positive control, the infected cells were treated with acyclovir (ACV, 2 µM) or treated with DMSO as negative control (CV). The results presented in all panels were analyzed with Bonferroni post-test correction for multiple comparisons. *** *p* < 0.001, and **** *p* < 0.0001 versus calibrator sample (CV).

Another biological effect that we evaluated was the antiviral activity against the clinical isolate of the HSV-1 virus. This is a widely spread herpetic virus that is relevant from a clinical and dermatological point of view, for which the available drugs (acyclovir, famciclovir, and valacyclovir) are characterized by numerous adverse effects, such as neurotoxicity and nephrotoxicity, and by poor bioavailability and effectiveness [40]. An additional important limitation associated with the current available drugs is the ability of HSV-1 to mutate and generate drug-resistant strains [41]. Therefore, because of the somewhat reduced clinical benefits of the above-mentioned drugs, new therapeutic alternatives are needed. The experimental approach we adopted was to evaluate the antiviral activity of DM samples against HSV-1, but as a preliminary test, any potential direct toxicity of DM samples on VERO cells was ascertained to exclude their cell toxicity on target cells. All three of the bioactive peptide samples (namely CtlP, LrP, and LcP) resulted to not be toxic for VERO cells, even at the highest concentration of 50 µg mL^−1^ (Table S2); thus, this concentration was used to assess their antiviral activity. Some wells were infected and treated with DMSO and used as a control to ascertain that the effect was not dependent on the solvent used to suspend the peptides. As can be seen in Figure 3, LrP, LcP, and CtlP are able to inhibit the replication of HSV-1 by more than 50%.

Similar to what was observed for the antibacterial activity, the inhibitory effect on the virus was stronger when the peptide mixture from DM fermented with *L. lactis* subsp. *cremoris* was used than for the *L. rhamnosus* fermented samples. However, a certain degree of variation was observed for both treatments, which was probably due to the intrinsic nature of the fermentation process, which is characterized by a high variability. Interestingly, the bioactive proteins or peptides present in the unfermented DM showed viral inhibition rates that achieved values of approximately 80–90%, thus suggesting that DM also possesses a natural antiviral activity against HSV-1. This had previously been demonstrated by Brumini et al. [42], who found that DM can inhibit the replication of the enterovirus Echovirus type 5, which is responsible for a wide variety of neurological and exanthematic diseases. These authors also demonstrated that this activity is due to high molecular weight whey proteins, such as lactoferrin, lactoperoxidase and immunoglobulin. Taken as a whole, the present results show an antiviral potential of the CtlP, LrP, and LcP samples that deserves further analyses. Although their activity against HSV-1 appears to show differences between the samples, such differences are not statistically significant. Since we ascertained that this antiviral property is not due to the cytotoxic effect on the cells by the peptide mixtures, it is possible to conclude that it depends on the peptide activity, although the action mechanism needs to be further investigated in more depth.

### 2.6. Characterization of the Peptide Profile of the Donkey Milk Samples

**Figure 4:**
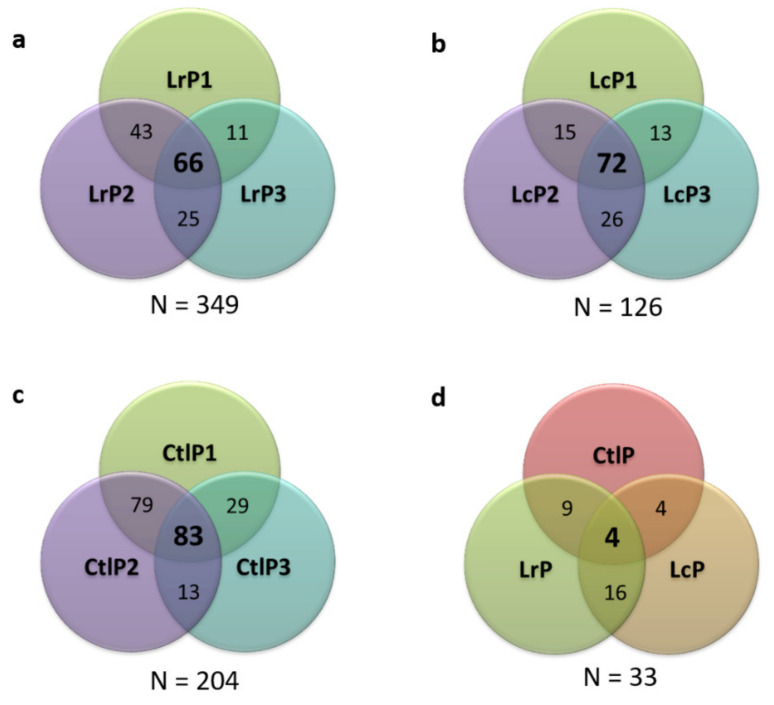
Venn diagrams showing the distribution of the identified peptides in the three biological replicates of each condition: (**a**) LrP, (**b**) LcP, (**c**) CtlP, and (**d**) comparing the three different tested conditions.

The analysis of the DM samples, by means of LC-MS/MS, provided 679 different peptide sequences, that is, 349 for the LrP samples, 126 for the LcP samples, and 204 peptides for the CtlP samples (Figure 4a–c). The Venn diagram related to all the tested conditions (Figure 4d) shows a small number of common peptides when the three conditions are compared, thus suggesting that the bacterial proteolytic system of each strain contributes to the DM peptidome composition differently. This evidence also supports the differences in terms of bioactivity observed among the samples, as previously described for both antimicrobial and antiviral activities.

**Figure 5:**
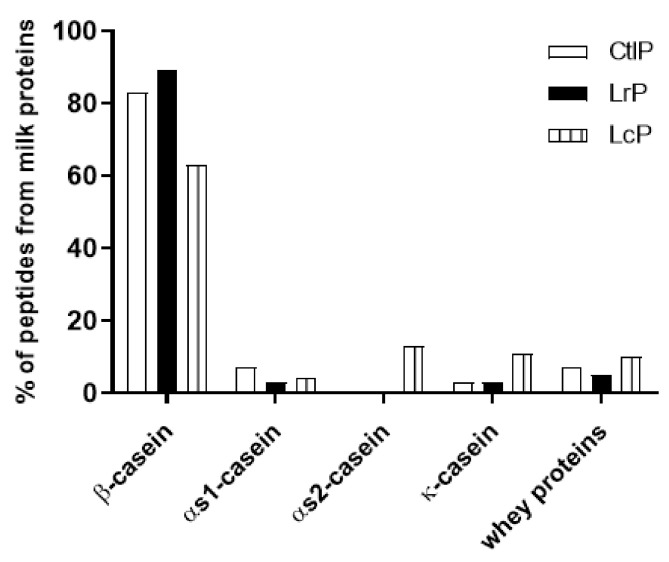
Histogram showing the main milk proteins from which the peptides from donkey milk inoculated with *L. rhamnosus* 17D10 (LrP), *L. lactis* subsp. *cremoris* 40FEL3 (LcP), and not inoculated (CtlP) were decrypted.

The evaluation of the proteins from which the peptides were mainly decrypted shows that β-casein is the most hydrolyzed protein, with 89%, 63%, and 83% of the peptides originated from it for the LrP, LcP, and CtlP samples, respectively (Figure 5). This result is not surprising, since β-casein is one of the most abundant proteins in DM [43], where it reaches about 50% of the caseins and 28% of the total protein content (casein and whey proteins). Moreover, it is possible to exclude that the identified encrypted peptides were from bacterial proteins, since no peptides were found when referring to the databases for *L. rhamnosus* and *L. lactis* subsp. *cremoris* (in addition to *Equus asinus*) for peptide matching.

The in silico analysis of the potential bioactivity of the identified peptides was performed using the free Milk Bioactive Peptide Database (MBPD) tool, setting the similarity threshold to 60% and identity as the scoring matrix. The results reported in Table 2 show that 41 of the bioactive peptides included in the database met the search criteria. β-casein, with 31 peptides, and κ-casein, with 10 peptides, are the two milk proteins from which all the bioactive peptides found in this research originated. The limit of this analysis is that the peptides from *Equus asinus* are poorly represented in MBPD compared to those from *Bos taurus*, due to the lack of an extensive scientific literature concerning bioactive peptides from DM. Moreover, as reported in Table 2, the maximum observed sequence similarity is 70% when the scoring matrix is set to identity. This result is not surprising, considering the differences in the primary structures of cow, human, and donkey milk proteins and the higher length shown by the peptides in this study than those of the peptides present in the database. A higher percentage of similarity, that is, of up to 80% was achieved for FIAIPPKK and VVPYPQRDTPVQAF when the scoring matrix was set to BLOSUM62, which matches each amino acid using blastp and a protein alignment substitution matrix. This result highlights how DM peptides show some amino acid substitutions, compared to peptides from *Bos taurus* and *Homo sapiens*, that involve amino acids with similar chemical properties, and therefore, these peptides could exert the same activity.

The DPATQPIVPVHNP, PATQPIVPVHNPV, and PATQPIVPVHNPVI peptides from the LrP sample, the FDPATQPIVPVHNPV one from the LcP sample, and PATQPIVPVHNPVIV identified in all the three DM samples show 60% similarity with the YPVTQPLAPVHNPIS antimicrobial peptide from *Homo sapiens*. This peptide, named CAMP211-225, is an endogenous peptide released from β-casein and is present in high concentrations in human milk from mothers who deliver preterm infants. Its antimicrobial activity was evaluated against six pathogenic bacterial strains—that is, *E. coli*, *Y. enterocolitica*, *L. monocytogenes*, *K. pneumoniae*, *S. aureus,* and *B. subtilis*. CAMP211-225 only inhibited the growth of *E. coli* and *Y. enterocolitica*, even for low concentrations (3–6 μg/mL), but it failed to affect the growth of the other tested bacteria, even for a high concentration (50 μg/mL) [44].

The VLPVPQKAVPYPQR antimicrobial peptide released from *Bos taurus* β-casein showed 60% similarity with the KVAPFPQPVVPYPQ and SKVAPFPQPVVPYPQ peptides from LrP and LcP, and with VAPFPQPVVPYPQ from LcP and VAPFPQPVVPYPQR from the CtlP samples. This antimicrobial peptide was found to be able to affect the growth of both Gram-positive (*S. aureus*) and Gram-negative (*E. coli*) bacteria, but it was less effective against *E. coli* [45].

Considering the evidences on the different behavior of the three biological replicates (LrP1, LrP2, and LrP3) to inhibit *E. coli* growth (Figure 2b), the in silico analyses of the peptides identified in the most active extract LrP3 was performed. Interestingly, we found a peptide PRIVLTPWDQTKT originated from alpha-S2-casein showing the 60% similarity to the antimicrobial peptide IVLNPWDQVK from *Bos taurus*. As reported by Liu et al. [46], this peptide showed antibacterial activity against *E. coli* NEB5, *E. coli* ATCC25922, and *B. subtilis* ATCC6051. The presence of a similar peptide from DM could explain the higher inhibitory effect of LrP3 extract compared to the other biological replicates.

As far as other activities are concerned, no peptide matching with peptides displaying antioxidant activity was found, in spite of the positive results obtained in this study when the antioxidant potential of the treated and untreated DM was evaluated as DPPH scavenging activity. A possible reason for this could be that the antioxidant peptides from DM have a lower similarity than 60% with those registered in the MBPDP. Thirteen peptides that matched the experimental peptide sequences showed ACE-inhibitory activity. On the other hand, an anti-thrombin peptide from cow κ-casein (MAIPPKK) was found to have 60% similarity with the FIAIPPKKLQ and FIAIPPKK peptides found in the LrC and CtlP samples, respectively, while the RDTPVQAFLLYQDPQLGLT peptide from LrP showed 60% similarity with an anti-inflammatory peptide from cow β-casein.

## 3. Materials and Methods

### 3.1. Donkey Milk

Donkey milk (DM) was obtained from a small dairy donkey farm located in San Benigno Canavese (TO). Immediately after collection, DM samples were stored at −20 °C and lyophilized (5Pascal, Trezzano sul Naviglio, Italy). Before lyophilization, raw milk was sampled for the microbiological characterization. The milk samples that were used for the experiments were representative of a pool of donkeys of different races, ages, and breastfeeding phases.

### 3.2. Microbial Strains and Growth Conditions

Bacteria from DM were isolated on Luria–Bertani (LB), Wallerstain (WL), Brain–Heart Infusion (BHI), DeMan–Rogosa–Sharpe (MRS), and M-17 agar plates (Sigma-Aldrich, St. Louis, MI, USA) at 37 °C for 24 h. *Lactobacillus reuteri* Lb2 BM-DSM, *Lactobacillus helveticus* 6E8, and *Lactobacillus rhamnosus* 17D10 bacteria, which were selected from the laboratory culture collection, were cultivated in MRS broth. *Lactococcus lactis* subsp. *cremoris* 40FEL3 and *Lactococcus lactis* subsp *lactis* MG1363 bacteria were cultivated in M-17 broth. All the tested strains, which were collected from fresh cultures at the beginning of the stationary phase, were maintained at −20 °C in 0.5 mL aliquots with 0.5 mL of 40% (*v*/*v*) glycerol. *Escherichia coli* and *Staphylococcus aureus*, which were used as target strains to evaluate the antimicrobial activity, were cultivated in LB broth.

### 3.3. Characterization of the Endogenous Microflora of the Donkey Milk

Cultivable mesophilic bacteria were isolated, at 37 °C, on LB, WL, BHI, MRS, and M-17 media. Colonies that showed a different morphology were selected. A pure solid sub-culture was obtained for each selected colony and was submitted to Gram staining and microscopy observation. Gram-positive bacteria were identified by means of the API 20 strep test, while Gram-negative bacteria were identified by means of API E and NE tests (bioMerieux SA, Lyon, France). Any bacteria that were not clearly identified or which showed an unacceptable profile after the API test were further investigated by means of RNA 16s analysis by colony PCR. The RNA 16s amplification was obtained using Phusion High-Fidelity DNA polymerase (BioLabs, Ipswich, MA, USA) according to the manufacturer’s instructions. Forward and reverse oligonucleotide universal primers (Eurofins Scientific), which hybridized to bacterial 16s rRNA, were used: 27f (5′-AGA GTT TGA TCA TGG CTC A-3′) and 1492r (5′-TAC GGT TAC CTT GTT ACG ACT T-3′). The amplification product was purified by means of the GeneElute PCR Clean-Up kit (Sigma Aldrich s.r.l.), and 16s rRNA was sequenced by the Eurofins Genomics service (Ebersberg, Germany). Sequence information was acquired by aligning the sequencing results with the sequences in Gene Bank using the BLAST search program of the National Center for Biotechnology Information (NCBI) (http://www.ncbi.nlm.nih.gov, accessed on 25 March 2017).

### 3.4. Selection of the LAB Strains for the Proteolytic Activity

Lyophilized DM was treated for 30 min at 85 °C immediately before each experiment, and after water solubilization, it was pasteurized for 30 min at 63 °C, with rapid cooling at 37 °C. Five food-isolated LAB strains were tested for their ability to grow in DM. The tested bacteria were *L. reuteri* Lb2 BM-DSM, *L. helveticus* 6E8, *L. rhamnosus* 17D10, *L. lactis* subsp. *cremoris* 40FEL3, and *L. lactis* subsp *lactis* MG1363. The selected LABs were grown in DM for 24 h at 37 °C under mild agitation to avoid casein precipitation. Bacterial growth and proteolytic activity were assessed by (i) measuring the pH of the culture to detect acidification, (ii) quantifying the L-lactate produced during fermentation (d-/l-Lactic Acid Assay Kit Megazyme Neogen (Lansing, MI, USA) according to the manufacturer’s instructions), and (iii) quantifying the released peptides by means of an OPA assay, using glycine as the standard.

### 3.5. Peptide Production from Donkey Milk by Selected Strains

*L. rhamnosus* 17D10 and *L. lactis* subsp. *cremoris* 40FEL3 were selected to perform fermentation experiments. The cell pellet of an o/n culture, with an OD600 nm of 0.100, was suspended in 1 mL of pasteurized DM for each bacterium, in order to avoid contamination of the milk with other medium components. Then, this bacterial suspension was inoculated in 50 mL of pasteurized DM and incubated for 24 h at 37 °C under mild agitation. Uninoculated DM was used as the control.

### 3.6. Peptide Extraction and Purification

Cell-free supernatants were collected, by means of centrifugation, for the DM fermented by LABs and for the non-fermented DM (control), (4000× *g*, 20 min, 4 °C) and filtered (0.22 µm filter, Millipore, Burlington, MA, USA). The supernatant was split into 2 mL acetone-compatible Eppendorf tubes, and four volumes of cold (−20 °C) acetone were added. The tubes were vortexed and incubated o/n at −20 °C. The supernatants were collected, by means of centrifugation (9400× *g*, 10 min at 4 °C), and dried using a vacuum concentrator (Christ RVC-2-18, Osterode am Harz, Germany). The dried pellets were resuspended in ddH20 0.1% TFA. Samples were purified, by means of SPE, on a reverse phase column (Phenomenex, Strata-X) as reported by Piovesana et al. [21]. Briefly, after conditioning the column with acetonitrile (ACN), peptides were rinsed with a 0.1% TFA aqueous solution and then eluted with ACN/ddH2O (70/30, *v*/*v*) with 0.1% TFA, and dried in the vacuum concentrator.

### 3.7. Evaluation of the Peptide Bioactivities

Dried peptides from DM fermented with *L. rhamnosus* 17D10 (LrP), with *L. lactis* subsp. *cremoris* 40FEL3 (LcP) and non-fermented DM (CtlP) were suspended in methanol for the antioxidant assay, in water for the metal chelating assay, and in a sterile solution for the antibacterial and the antiviral assays. Three replicates of the activity assays were performed for each peptide mixture.

#### 3.7.1. Antioxidant Activity

The DPPH scavenging activity of the DM peptide extract was evaluated using the Amarowicz et al. [47] method. Five dilutions of the peptide samples (0–100 µL) were mixed with 2 mL of a 0.125 mM DPPH methanol solution. The mixture was vortexed for 1 min, left to stand at room temperature for 20 min, and then, the absorbance of the resulting solutions was read at 517 nm. The antioxidant properties were expressed as IC50 values, which were defined as the concentration of the extract needed to scavenge 50% of the initial DPPH.

#### 3.7.2. Metal-Chelating Activity

The iron and copper chelating activities were evaluated according to the methods reported by Durak et al. [48].

#### 3.7.3. Antibacterial Activity

The antibacterial activity was evaluated in 96-well plates as the means of growth inhibition of two representative cell types, that is, the Gram-negative *E. coli* strain and the Gram-positive *S. aureus* strain in an LB medium vs. pathogen growth. Pathogen growth in the presence of DM peptides, tested at the single concentration of 25 µg mL^−1^, was monitored for 24 h at 37 °C by measuring OD595 (Filter Max F5).

#### 3.7.4. Antiviral Activity

The potential cytotoxic effects of the peptides derived from the proteolytic activity of LrP, LcP, and CtlP were evaluated on VERO cells (African green monkey kidney cells, ATCC-American Type Culture Collection CCL-81) [49], as described in Bovio et al. [50] The peptides were resuspended in DMSO at a concentration of 10 mg mL^−1^. In order to determine cell viability, the confluent VERO cells were exposed to increasing concentrations of the peptides (12.5, 25.0, or 50.0 μg mL^−1^), the plate was incubated for 72 h at 37 °C. The number of viable cells was determined using the CellTiter-Glo Luminescent assay (Promega), as described in Luganini et al. [51]. The peptides were assessed for their antiviral activity at a concentration that produced at least 70% cell viability. VERO cells were seeded in 24-well plates, at a density of 6X104 cells per well, for a plaque reduction assay (PRA) with the HSV-1 virus (acyclovir-sensitive herpes simplex virus 1 clinical isolate, kindly provided by V. Ghisetti, Amedeo di Savoia Hospital, Turin, Italy). The following day, the cultures were treated with 50.0 μg mL^−1^ of each peptide 1 h prior to infection and then infected with 45 plaque-forming units (PFU)/well in the presence of the compounds. Following virus adsorption (2 h at 37 °C), cultures were maintained in medium containing the corresponding compounds, supplemented with 3% FBS and 0.9% Avicel [50]. The procedure was performed in duplicate in three independent experiments. Control wells with mock-infected cells and untreated virus-infected cells were included in each plate. At 48 h post-infection (p.i.), cell monolayers were fixed with 4% formaldehyde for 1 h at room temperature (RT) and stained with a 1% crystal violet solution for 40 min before the viral plaques were microscopically counted. The mean plaque counts of each compound were expressed as a percentage of the mean plaque count of the control virus.

### 3.8. Peptide Identification by Means of LC-MS/MS Analysis

LC–MS/MS analyses were performed using a micro-LC Eksigent Technologies (Dublin, CA, USA) system with a stationary phase of a Halo Fused C18 column (0.5 × 100 mm, 2.7 μm; Eksigent Technologies, Dublin, USA). The injection volume was 4.0 μL, and the oven temperature was set at 40 °C. The mobile phase was a mixture of 0.1% (*v*/*v*) formic acid in water (A) and 0.1% (*v*/*v*) formic acid in acetonitrile (B), which was eluted at a flow rate of 15.0 μL min^−1^ with increasing concentrations of B, ranging from 2% to 40%, over 30 min. The peptides were suspended in 10 μL of A. The LC system was interfaced with a 5600+ TripleTOF system (AB Sciex, Concord, ON, Canada) equipped with a DuoSpray Ion Source and a CDS (Calibrant Delivery System). Peptide identification was performed using the traditional data-dependent acquisition (DDA) method. The MS data were acquired with Analyst TF 1.7 (SCIEX, Concord, ON, Canada). The mass spectrometry proteomics data have been deposited to the ProteomeXchange Consortium via the PRIDE [52] partner repository (https://www.ebi.ac.uk/pride/archive/, accessed on 8 August 2021) with the dataset identifier PXD027765.

### 3.9. Peptide Data Searc

The MS files were searched using Mascot software, v. 2.4 (Matrix Science Inc., Boston, MA, USA) setting no enzyme as the digest enzyme, 50 ppm as the peptide mass tolerance, 0.1 Da as the MS/MS tolerance, and 2+, 3+, and 4+ as the peptide charges; the search was set on the monoisotopic mass. The instrument was set to ESI-QUAD-TOF. The UniProt/Swiss-Prot reviewed database containing *Equus asinus*, *L. rhamnosus*, and *L. lactis* subsp. *cremoris* proteins (NCBI *Equus asinus*, version 23092019, 42,662 sequences; NCBI *Lactobacillus rhamnosus*, version 23092019, 425,126 sequences; *Lactobacillus lactis* subsp. *cremoris* 23092019, 147,917 sequences) was used. Only the peptides identified with a higher peptide score than the peptide identity were considered for each biological replicate. Moreover, only the peptides that were common to the three biological replicates were taken into account for further data elaboration.

### 3.10. Peptide in Silico Analyses

The peptide sequences shared by the three replicates of each condition were evaluated for their potential bioactivity using the online Milk Bioactive Peptide DataBase (MBPDB) (http://mbpdb.nws.oregonstate.edu/, accessed on 25 July 2021) [53]. The similarity was evaluated for the scoring matrix, identity, and BLOSUM 62, and the threshold was set to a range of 60–100%.

### 3.11. Statistical Analysis

All the statistical tests were performed using GraphPad Prism, version 5.0, for Windows. The data are presented as the means ± SDs of at least three duplicated experiments.

## 4. Conclusions

In the present study, the fermentation of donkey milk with selected LAB strains has been shown to increase the release of bioactive peptides. In such context, it could be interesting to set up protocols aimed at selecting the most effective fermentative conditions in order to increase the concentrations of these bioactive compounds. Although donkey milk is already a well-established nutraceutical, the LAB fermentation process could enhance its value, thus making this product an interesting fermented functional food. In short, the results of this study confirm that food fermentation is a promising and cheap strategy to generate bioactive peptides, especially when a bioactive food matrix is combined with the probiotic properties of LABs.

## Figures and Tables

**Table 1:** Quantification of released peptides, L-lactic acid content, and pH after 24 h donkey milk fermentation by the five selected LAB strains.

	Peptide (mg mL^−1^)	L-Lactic Acid (mg mL^−1^)	pH
*L. reuteri* Lb2 BM-DSM	0.082 ± 0.005	0.568 ± 0.033	6.27 ± 0.29
*L. helveticus* 6E8	0.107 ± 0.014	0.813 ± 0.019	5.21 ± 0.31
*L. rhamnosus* 17D10	0.283 ± 0.008	4.501 ± 0.107	3.86 ± 0.19
*L. lactis* subsp *cremoris* 40FEL3	0.221 ± 0.015	3.390 ± 0.099	4.24 ± 0.28
*L. lactis* subsp *lactis* MG1363	0.170 ± 0.006	0.219 ± 0.023	6.33 ± 0.25

**Table 2:** List of the peptides from donkey milk inoculated with *L. rhamnosus* 17D10 (LrP), *L. lactis* subsp. *cremoris* 40FEL3 (LcP), and not inoculated (CtlP) showing at least 60% of similarity (identity as scoring matrix) to the bioactive peptides present in the Milk Bioactive Peptide Database (MBPD).

Experimental Peptide	Sample	% Similarity (Identity/Blosum 62)	Peptide from Database	Specie	Protein	AA Intervals	Activity
DPATQPIVPVHNP	LrP	60/60	YPVTQPLAPVHNPIS	*Homo sapiens*	β-casein	211–225	Antimicrobial
FDPATQPIVPVHNPV	LcP	60/70	YPVTQPLAPVHNPIS	*Homo sapiens*	β-casein	211–225	Antimicrobial
FIAIPPKK	CtlP	70/80	MAIPPKK	*Bos taurus*	κ-casein	127–133	Antithrombin ACE-inhibitory
		60/60	IAIPP	*Homo sapiens*	κ-casein	118–122	ACE-inhibitory
		60/60	AIPPKKNQD	*Bos taurus*	κ-casein	128–136	ACE-inhibitory
FIAIPPKKLQ	LrP	70/70	AIPPKKNQD	*Bos taurus*	κ-casein	128–136	ACE-inhibitory
		60/70	MAIPPKK	*Bos taurus*	κ-casein	127–133	Antithrombin
KVAPFPQPVVPYPQ	LrP, LcP	70/70	VAPFPQPVVP	*Equus asinus*	β-casein	176–185	ACE-inhibitory
		60/60	VLPVPQKAVPYPQR	*Bos taurus*	β-casein	185–198	Antimicrobial
LPSQPVLSPPQSKVAPFP	LrP	60/60	PPQSVLSLSQSKVLPVPQ	*Bos taurus*	β-casein	173–190	ACE-inhibitory
PATQPIVPVHNPV	LrP	60/70	YPVTQPLAPVHNPIS	*Homo sapiens*	β-casein	211–225	Antimicrobial
PATQPIVPVHNPVI	LrP	60/70	YPVTQPLAPVHNPIS	*Homo sapiens*	β-casein	211–225	Antimicrobial
PATQPIVPVHNPVIV	LrP, LcP, CtlP	60/70	YPVTQPLAPVHNPIS	*Homo sapiens*	β-casein	211–225	Antimicrobial
QPVVPYPQRDTPVQAF	CtlP	60/70	VPYPQRDMPIQAFL	*Bos taurus*	β-casein	193–206	Antimicrobial
QSKVAPFPQPVVPYPQ	LrP	60/70	VAPFPQPVVP	*Equus asinus*	β-casein	176–185	ACE-inhibitory
RDTPVQAFLL	LrP	60/70	RDMPIQAF	*Bos taurus*	β-casein	198–205	ACE-inhibitory
RDTPVQAFLLYQDPQLGLT	LrP	60/70	DMPIQAFLLYQEPVLGPVR	*Bos taurus*	β-casein	199–217	Anti-inflammatory
SKVAPFPQPVVPYPQ	LrP, LcP	60/60	VLPVPQKAVPYPQR	*Bos taurus*	β-casein	185–198	Antimicrobial
			VAPFPQPVVP	*Equus asinus*	β-casein	176–185	ACE-inhibitory
VAPFPQPVVPYPQ	LcP	70/70	VAPFPQPVVP	*Equus asinus*	β-casein	176–185	ACE-inhibitory
		60/70	VLPVPQKAVPYPQR	*Bos taurus*	β-casein	185–198	Antimicrobial
VAPFPQPVVPYPQR	CtlP	70/70	VLPVPQKAVPYPQR	*Bos taurus*	β-casein	185–198	Antimicrobial
		70/70	VAPFPQPVVP	*Equus asinus*	β-casein	176–185	ACE-inhibitory
VVPYPQRDTPVQAF	CtlP	70/80	VPYPQRDMPIQAFL	*Bos taurus*	β-casein	193–206	Antimicrobial

## Data Availability

The data presented in this study are contained within the article.

## Supplementary Materials

The following is available online, Table S1: List of the peptides identified by means of LC-MS/MS from donkey milk fermented with *L. rhamnosus* 17D10 (LrP), *L. lactis* subsp. *cremoris* 40FEL3 (LcP) and not inoculated donkey milk (CtlP). Only the peptides shared by the three biological replicates are listed in the table for each condition. Table S2. Cell viability at different concentrations of peptides from donkey milk against VERO cells. The table contains the data of cytotoxicity at different concentrations (12.5 µg mL^−1^, 25 µg mL^−1^, 50 µg mL^−1^) of peptides from donkey milk inoculated with L. rhamnosus 17D10 (LrP), L. lactis subsp. cremoris 40FEL3 (LcP), and not fermented (CtlP), as determined by cell viability assays in VERO cells at 72 h post-treatment. The experiment was performed in triplicate for each of the three biological replicates of each extract (LrP, LcP and CtlP). The percentage of cell viability were calculated by comparison with the mock control, which corresponds to the cells treated with the vehicle (DMSO).

## Sample Availability

Samples of the compounds are not available from the authors.
