# Supplementary material for: Donkey Milk Fermentation by Lactococcus lactis subsp. cremoris and Lactobacillus rhamnosus Affects the Antiviral and Antibacterial Milk Properties
Source: Molecules. 2021 Aug 23;26(16):5100. doi: 10.3390/molecules26165100 (PMC8398202; doi:10.3390/molecules26165100)
Supplement: Supplementary file 1 [file molecules-26-05100-s001.zip › molecules-1298494-supplementary.pdf]

Table S1. List of the peptide identified by means of LC-MS/MS from donkey milk fermented with *L. rhamnosus* 17D10 (LrP), *L. lactis* subsp. *cremoris* 40FEL3 (LcP) and not inoculated donkey milk (CtlP). Only the peptides shared by the three biological replicates are listed in the table for each condition.

SAMPLE	PEPTIDE SEQUENCE	PROTEIN ACCESSION	DESCRIPTION
CtlP	ALQPLPGRVQIIPDLTR	XP_014693525.1	beta-lactoglobulin-1
CtlP	AVVPQNILPLAQPPIVP	XP_014708641.1	beta-casein
CtlP	DKNGVTP	XP_014714975.1	E3 ubiquitin-protein ligase HACE1
CtlP	DPATQPIVPVHNPVIV	XP_014708641.1	beta-casein
CtlP	FALEYINELNR	XP_014708642.1	alpha-S1-casein
CtlP	FDPATQPIVPVHNPVIV	XP_014708641.1	beta-casein
CtlP	FIAIPPKK	XP_014702750.1	kappa-casein
CtlP	FIITLRNF	CAX65660.2	alpha-S2-casein
CtlP	FLALHVLT	XP_014683056.1	epithelial chloride channel protein-like
CtlP	FLKSPIVPFSERQ	XP_014708641.1	beta-casein
CtlP	FLKSPIVPFSERQI	XP_014708641.1	beta-casein
CtlP	FLKSPIVPFSERQILNPTNGENLRLP	XP_014708641.1	beta-casein
CtlP	FLKSPIVPFSERQILNPTNGENLRLPVH	XP_014708641.1	beta-casein
CtlP	FLLTLSTQT	XP_014693013.1	tumor necrosis factor receptor superfamily member 21
CtlP	FLLYQDPQ	XP_014708641.1	beta-casein
CtlP	FLLYQDPQLG	XP_014708641.1	beta-casein
CtlP	FLLYQDPQLGL	XP_014708641.1	beta-casein
CtlP	FLLYQDPQLGLTGEFD	XP_014708641.1	beta-casein
CtlP	FLLYQDPQLGLTGEFDPATQPIVP	XP_014708641.1	beta-casein
CtlP	FLLYQDPQLGLTGEFDPATQPIVPVHNP	XP_014708641.1	beta-casein
CtlP	FLLYQDPQLGLTGEFDPATQPIVPVHNPVIV	XP_014708641.1	beta-casein
CtlP	FLQPEIMEVSQAK	XP_014708641.1	beta-casein
CtlP	FLQPEIMEVSQAKETILPK	sp|P86273.1|CASB_EQUAS	Beta-casein
CtlP	FLQPEIMEVSQAKETILPKRKVMP	sp|P86273.1|CASB_EQUAS	Beta-casein
CtlP	FLQPEIMEVSQAKETLLPK	XP_014708641.1	beta-casein
CtlP	FLQPEIMEVSQAKETLLPKRK	XP_014708641.1	beta-casein
CtlP	FLQPEIMEVSQAKETLLPKRKVMP	XP_014708641.1	beta-casein
CtlP	FMHQVPQSLLQT	XP_014708641.1	beta-casein
CtlP	FMHQVPQSLLQTL	XP_014708641.1	beta-casein
CtlP	FMHQVPQSLLQTLMLPSQP	XP_014708641.1	beta-casein
CtlP	FSERQILNPTNGENLRLPVH	XP_014708641.1	beta-casein
CtlP	FYLEPFQP	XP_014708642.1	alpha-S1-casein isoform X1
CtlP	ILNPTNGENLRLPVH	XP_014708641.1	beta-casein
CtlP	ILPLAQPPIVP	XP_014708641.1	beta-casein
CtlP	ILPLAQPPIVPFL	XP_014708641.1	beta-casein
CtlP	ILPLAQPPIVPFLQPEIMEVSQAKET	XP_014708641.1	beta-casein
CtlP	ILPLAQPPIVPFLQPEIMEVSQAKETLLPKRK	XP_014708641.1	beta-casein
CtlP	IVPFSERQILNPTNGENLRLPVH	XP_014708641.1	beta-casein
CtlP	IYAQEQLLR	XP_014708642.1	alpha-S1-casein isoform X1
CtlP	LAQPPIVPFLQPEIMEVSQAKET	sp|P86273.1|CASB_EQUAS	Beta-casein
CtlP	LAQPPIVPFLQPEIMEVSQAKETLLPKRK	XP_014708641.1	beta-casein
CtlP	LGLTGEFDPATQPIVPVHNPVIV	XP_014708641.1	beta-casein
CtlP	LKSPIVPFSER	XP_014708641.1	beta-casein
CtlP	LLYQDPQLGLTGEFDPATQPIVPVHNP	XP_014708641.1	beta-casein
CtlP	LLYQDPQLGLTGEFDPATQPIVPVHNPVIV	XP_014708641.1	beta-casein

CtlP	LMLPSQPVLSPPQSK	XP_014708641.1	beta-casein
CtlP	LNPTNGENLRLP	XP_014708641.1	beta-casein
CtlP	LNPTNGENLRLPVHL	XP_014708641.1	beta-casein
CtlP	LQAIYAQEQLLR	XP_014708642.1	alpha-S1-casein isoform X1
CtlP	LRLPVHL	XP_014708641.1	beta-casein
CtlP	LRLPVHLIQP	XP_014708641.1	beta-casein
CtlP	LTGEFDPATQPIVP	XP_014708641.1	beta-casein
CtlP	LTGEFDPATQPIVPVH	XP_014708641.1	beta-casein
CtlP	LTGEFDPATQPIVPVHN	XP_014708641.1	beta-casein
CtlP	LTGEFDPATQPIVPVHNP	XP_014708641.1	beta-casein
CtlP	LTGEFDPATQPIVPVHNPVIV	XP_014708641.1	beta-casein
CtlP	LVIVPNMLA	XP_014692308.1	olfactory receptor 10V1-like
CtlP	LVKINPKFPSPQY	CAX65660.2	alpha s2 casein B
CtlP	LYQDPQLGLTGEFDPATQPIVPVHNP	XP_014708641.1	beta-casein
CtlP	LYQDPQLGLTGEFDPATQPIVPVHNPVIV	XP_014708641.1	beta-casein
CtlP	NLRLPVHL	XP_014708641.1	beta-casein
CtlP	NPTNGENLRLPVH	XP_014708641.1	beta-casein
CtlP	PATQPIVPVHNPVIV	XP_014708641.1	beta-casein
CtlP	PIVPVHNPVIV	XP_014708641.1	beta-casein
CtlP	PPIVPFLQPEIMEVSQAKET	XP_014708641.1	beta-casein
CtlP	PQLGLTGEFDPATQPIVP	XP_014708641.1	beta-casein
CtlP	PQNILPLAQPPIVPF	XP_014708641.1	beta-casein
CtlP	PYAVVPQNILPLAQPPIVP	XP_014708641.1	beta-casein
CtlP	QILNPTNGENLRLP	XP_014708641.1	beta-casein
CtlP	QILNPTNGENLRLPVHL	XP_014708641.1	beta-casein
CtlP	QLGLTGEFDPATQPIVPVHNPVIV	XP_014708641.1	beta-casein
CtlP	QPVVPYPQRDTPVQAF	XP_014708641.1	beta-casein
CtlP	SPIVPFSERQ	XP_014708641.1	beta-casein
CtlP	SPPQSKVAPFPQPVVPYPQRDTPVQA	XP_014708641.1	beta-casein
CtlP	TPVQAFLLYQDPQLGLTGEFDPATQPIVPVHNPVIV	XP_014708641.1	beta-casein
CtlP	VAPFPQPVVPYPQR	XP_014708641.1	beta-casein
CtlP	VAPFPQPVVPYPQRDTP	XP_014708641.1	beta-casein
CtlP	VLSPPGA	XP_014701853.1	antigen peptide transporter 2
CtlP	VMPFLKSPIVPFSERQILNPTNGENLRLPVH	XP_014708641.1	beta-casein
CtlP	VPYAVVPQNILPLAQPPIVPF	XP_014708641.1	beta-casein
CtlP	VQIPQWQVLPNIYPS	XP_014702750.1	kappa-casein
CtlP	VVPQNILPLAQPPIVP	XP_014708641.1	beta-casein
CtlP	VVPYPQRDTPVQAF	XP_014708641.1	beta-casein
LcP	AAWFHPAQIMQH	1::sp|P86272.1|CASA1_EQUAS	Alpha-S1-casein
LcP	AAWFHPAQIMQHVAYSPFHDTAKLIASEN	1::sp|P86272.1|CASA1_EQUAS	Alpha-S1-casein
LcP	AIYAQEQLIR	1::sp|P86272.1|CASA1_EQUAS	Alpha-S1-casein
LcP	ARPAAVRPHVQIPQWQVLPNIYPS	1::XP_014702750.1	kappa-casein
LcP	AVLVAAG	1::XP_014724157.1	dynein heavy chain 12, axonemal isoform X1
LcP	AVRPHVQIPQWQVLPN	1::XP_014702750.1	kappa-casein
LcP	AVRPHVQIPQWQVLPNIYPS	1::XP_014702750.1	kappa-casein
LcP	DPATQPIVPVHNPVIV	1::CBI63375.1	beta-casein
LcP	DPQLGLTGEFDPATQPIVPVHNPVIV	1::CBI63375.1	beta-casein
LcP	DQTKTGASPFIPIVN	1::sp|B7VGF9.1|CASA2_EQUAS	Alpha-S2-casein; Flags:
LcP	DVHKAVLVAQ	1::XP_014697927.1	osteopontin
LcP	DVHKAVLVAQGLHVA	1::XP_014697927.1	osteopontin
LcP	FDPATQPIVPVHNPV	1::CBI63375.1	beta-casein
LcP	FDPATQPIVPVHNPVIV	1::CBI63375.1	beta-casein

LcP	GENLRLPVHLIQPF	1::CBI63375.1	beta-casein
LcP	HDTAKLIASE	1::sp|P86272.1|CASA1_EQUAS	Alpha-S1-casein
LcP	HRLAVLINNQHMPYQYYARPA	1::XP_014702750.1	kappa-casein
LcP	HVAYSPFHDTAK	1::sp|P86272.1|CASA1_EQUAS	Alpha-S1-casein
LcP	ILNPTNGENLRLPVH	1::CBI63375.1	beta-casein
LcP	ISRFVQPQPVVYPY	1::CBI63375.1	beta-casein
LcP	KVAPFPQPVVPYPQ	1::CBI63375.1	beta-casein
LcP	LKMNILGLVLP	1::XP_014707907.1	C-C chemokine receptor type 1
LcP	LMLPSQPVLSPPQSK	1::CBI63375.1	beta-casein
LcP	LPSQPVLSPPQSK	1::CBI63375.1	beta-casein
LcP	LRLPVHLIQPF	1::CBI63375.1	beta-casein
LcP	LRLPVHLIQPFMH	1::CBI63375.1	beta-casein
LcP	MHQVPQSLLQT	1::CBI63375.1	beta-casein
LcP	MHQVPQSLLQTL	1::CBI63375.1	beta-casein
LcP	MHQVPQSLLQTLML	1::CBI63375.1	beta-casein
LcP	MHQVPQSLLQTLMLPSQPVL	1::CBI63375.1	beta-casein
LcP	MHQVPQSLLQTLMLPSQPVLSPPQ	1::CBI63375.1	beta-casein
LcP	MHQVPQSLLQTLMLPSQPVLSPPQSK	1::CBI63375.1	beta-casein
LcP	MLPSQPVLSPPQSK	1::CBI63375.1	beta-casein
LcP	MPFLKSPIVPF	1::CBI63375.1	beta-casein
LcP	NGENLRLPV	1::CBI63375.1	beta-casein
LcP	NGENLRLPVHLIQPF	1::CBI63375.1	beta-casein
LcP	NILPLAQPPIVPF	1::CBI63375.1	beta-casein
LcP	NLRLPVHLIQPF	1::CBI63375.1	beta-casein
LcP	NNQHMPYQYYARPA	1::XP_014702750.1	kappa-casein
LcP	NQLQLQAIYAQ	1::sp|P86272.1|CASA1_EQUAS	Alpha-S1-casein
LcP	PATQPIVPVHNPVIV	1::CBI63375.1	beta-casein
LcP	PIVPVHNPVIV	1::CBI63375.1	beta-casein
LcP	PPQSKVAPFPQPVVPYPQ	1::CBI63375.1	beta-casein
LcP	PQLGLTGEFDPATQPIVPVHNPVIV	1::CBI63375.1	beta-casein
LcP	PSQPVLSPPQSK	1::CBI63375.1	beta-casein
LcP	QDPQLGLTGEFDPATQPIVPVHNPVIV	1::CBI63375.1	beta-casein
LcP	RDTPVQAFLLY	1::CBI63375.1	beta-casein
LcP	RFVQPQPVVYPY	1::CBI63375.1	beta-casein
LcP	RKVMPFLKSPIVPF	1::CBI63375.1	beta-casein
LcP	RKVMPFLKSPIVPFSERQILNPTNGEN	1::CBI63375.1	beta-casein
LcP	RLAVLINNQHMPY	1::XP_014702750.1	kappa-casein
LcP	RLPVHLIQPF	1::CBI63375.1	beta-casein
LcP	RVVNQEQAY	1::sp|P86272.1|CASA1_EQUAS	Alpha-S1-casein
LcP	RVVNQEQAYF	1::sp|P86272.1|CASA1_EQUAS	Alpha-S1-casein
LcP	RVVNQEQAYFY	1::sp|P86272.1|CASA1_EQUAS	Alpha-S1-casein
LcP	SERQILNPTNGE	1::CBI63375.1	beta-casein
LcP	SERQILNPTNGEN	1::CBI63375.1	beta-casein
LcP	SKVAPFPQPVVPYPQ	1::CBI63375.1	beta-casein
LcP	SKVVVSTLVPLA	1::XP_014684365.1	polymeric immunoglobulin receptor
LcP	SPPQSKVAPFPQPVVPYPQ	1::CBI63375.1	beta-casein
LcP	SRFVQPQPVVYPY	1::CBI63375.1	beta-casein
LcP	TPVQAFLLYQDPQLGLTGEFDPATQPIVPVHNPVIV	1::CBI63375.1	beta-casein
LcP	VAPFPQPVVPYPQ	1::CBI63375.1	beta-casein
LcP	VHKAVLVAQ	1::XP_014697927.1	osteopontin
LcP	VHKAVLVAQGLHVA	1::XP_014697927.1	osteopontin
LcP	VKINPKFPSPQYFQ	1::CAX65660.2	alpha s2 casein

LcP	VKINPKFPSPQYFQA	1::CAX65660.2	alpha s2 casein
LcP	VQIPQWQVLPNIYPS	1::XP_014702750.1	kappa-casein
LcP	YARPAAVRPHVQIPQWQVLPNIYPS	1::XP_014702750.1	kappa-casein
LcP	YQDPQLGLTGE	1::CBI63375.1	beta-casein
LcP	YQDPQLGLTGEFDPA	1::CBI63375.1	beta-casein
LcP	YQDPQLGLTGEFDPATQPIVPVHNPVIV	1::CBI63375.1	beta-casein
LrP	APFPQPVVPYPQRDTP	1::CBI63375.1	beta-casein
LrP	AQPPIVPFLQPEIMEVSQAKETLLPKRKVMPFL	1::CBI63375.1	beta-casein
LrP	AVVPQNILPLAQPPIVP	1::CBI63375.1	beta-casein
LrP	DPATQPIVPVHNP	1::CBI63375.1	beta-casein
LrP	DPATQPIVPVHNPVIV	1::CBI63375.1	beta-casein
LrP	DPQLGLTGEFDPATQPIVPVHNPVIV	1::CBI63375.1	beta-casein
LrP	FIAIPPKKLQ	1::CBZ41787.1	kappa-casein
LrP	FLALHVLT	1::XP_014683056.1	epithelial chloride channel protein-like
LrP	GEFDPATQPIVPVH	1::CBI63375.1	beta-casein
LrP	GEFDPATQPIVPVHNPVI	1::CBI63375.1	beta-casein
LrP	GEFDPATQPIVPVHNPVIV	1::CBI63375.1	beta-casein
LrP	ILNPTNGENLRLP	1::CBI63375.1	beta-casein
LrP	ILNPTNGENLRLPVH	1::CBI63375.1	beta-casein
LrP	ILPLAQPPIVP	1::CBI63375.1	beta-casein
LrP	INPKFPSPQY	1::CAX65660.2	alpha s2 casein
LrP	IWLKPDPSQK	1::XP_014697927.1	osteopontin
LrP	KVAPFPQPVVPYPQ	1::CBI63375.1	beta-casein
LrP	LKSPIVPFSERQILNPTNGENLRLPVH	1::CBI63375.1	beta-casein
LrP	LNPTNGENLRLP	1::CBI63375.1	beta-casein
LrP	LNPTNGENLRLPVH	1::CBI63375.1	beta-casein
LrP	LPSQPVLSPPQSK	1::CBI63375.1	beta-casein
LrP	LPSQPVLSPPQSKVAPFP	1::CBI63375.1	beta-casein
LrP	LRLPVHLIQP	1::CBI63375.1	beta-casein
LrP	LRLPVHLIQPF	1::CBI63375.1	beta-casein
LrP	LVKINPKFPSPQY	1::CAX65660.2	alpha s2 casein
LrP	LYQDPQLGLTGEFDPATQPIVPVHNPVIV	1::CBI63375.1	beta-casein
LrP	MHQVPQSLLQTL	1::CBI63375.1	beta-casein
LrP	MHQVPQSLLQTLMLPSQP	1::CBI63375.1	beta-casein
LrP	MHQVPQSLLQTLMLPSQPVLSPPQ	1::CBI63375.1	beta-casein
LrP	MLPSQPVLSPPQSK	1::CBI63375.1	beta-casein
LrP	NILPLAQPPIVP	1::CBI63375.1	beta-casein
LrP	NLRLPVHLIQP	1::CBI63375.1	beta-casein
LrP	NLRLPVHLIQPF	1::CBI63375.1	beta-casein
LrP	NPTNGENLRLPV	1::CBI63375.1	beta-casein
LrP	PATQPIVPVHNPV	1::CBI63375.1	beta-casein
LrP	PATQPIVPVHNPVI	1::CBI63375.1	beta-casein
LrP	PATQPIVPVHNPVIV	1::CBI63375.1	beta-casein
LrP	PIVPVHNPVIV	1::CBI63375.1	beta-casein
LrP	PPQSKVAPFPQPVVPYPQ	1::CBI63375.1	beta-casein
LrP	PQLGLTGEFDPATQPIVPVHNPVIV	1::CBI63375.1	beta-casein
LrP	PTNGENLRLPVH	1::CBI63375.1	beta-casein
LrP	PYAEPVPYAVVPQNILP	1::CBI63375.1	beta-casein
LrP	QDPQLGLTGEFDPATQPIVPVHNPVIV	1::CBI63375.1	beta-casein
LrP	QSKVAPFPQPVVPYPQ	1::CBI63375.1	beta-casein
LrP	RDTPVQAFLL	1::CBI63375.1	beta-casein
LrP	RDTPVQAFLLY	1::CBI63375.1	beta-casein

LrP	RDTPVQAFLLYQDPQLGLT	1::CBI63375.1	beta-casein
LrP	RFVQPQPVVYP	1::CBI63375.1	beta-casein
LrP	RFVQPQPVVYPY	1::CBI63375.1	beta-casein
LrP	RFVQPQPVVYPYAEP	1::CBI63375.1	beta-casein
LrP	RFVQPQPVVYPYAEPVPY	1::CBI63375.1	beta-casein
LrP	RLPVHLIQP	1::CBI63375.1	beta-casein
LrP	RLPVHLIQPF	1::CBI63375.1	beta-casein
LrP	RQILNPTNGENLRLP	1::CBI63375.1	beta-casein
LrP	SKVAPFPQPVVPYPQ	1::CBI63375.1	beta-casein
LrP	SPIVPFSERQILNPTNGENLRLP	1::CBI63375.1	beta-casein
LrP	SPIVPFSERQILNPTNGENLRLPVH	1::CBI63375.1	beta-casein
LrP	SPPQSKVAPFPQPVVPYPQ	1::CBI63375.1	beta-casein
LrP	SPPQSKVAPFPQPVVPYPQR	1::CBI63375.1	beta-casein
LrP	SPPQSKVAPFPQPVVPYPQRDTPVQ	1::CBI63375.1	beta-casein
LrP	SSPRNEPIYYQHR	1::CBZ41787.1	kappa-casein
LrP	VHKAVLVAQ	1::XP_014697927.1	osteopontin
LrP	VLSPPQSKVAPFPQPVVPYPQR	1::CBI63375.1	beta-casein
LrP	VVPQNILPLAQPPIVP	1::CBI63375.1	beta-casein
LrP	YQDPQLGLTGEFDPATQPIVPVH	1::CBI63375.1	beta-casein
LrP	YQDPQLGLTGEFDPATQPIVPVHNP	1::CBI63375.1	beta-casein

Table S2. Cell viability at different concentrations of peptides from donkey milk against VERO cells. In the table are reported the data of cytotoxicity at different concentrations (12.5 μg mL^{-1}, 25 μg mL^{-1}, 50 μg mL^{-1}) of peptides from donkey milk inoculated with *L. rhamnosus* 17D10 (LrP), *L. lactis* subsp. *cremoris* 40FEL3 (LcP), and not fermented (CtlP), as determined by cell viability assays in VERO cells at 72 h post-treatment. The experiment was performed in triplicate for each of the three biological replicates of each extract (LrP, LcP and CtlP). The percentage of cell viability were calculated by comparison with the mock control, that corresponds to the cells treated with the vehicle (DMSO).

**Peptide concentration: 50 μg mL^{-1}**						
**Sample**	**Cell viability**			**Percentage of cell viability**		
**LrP1**	36533885	37553390	35772433	**95.18534**	**97.84156**	**93.20145**
**LrP2**	38039017	38775237	39488092	**99.10681**	**101.025**	**102.8822**
**LrP3**	39143523	37211438	37909358	**101.9845**	**96.95063**	**98.76899**
**LcP1**	39622462	38119854	35878028	**103.2323**	**99.31742**	**93.47657**
**LcP2**	38110826	38095043	43853680	**99.2939**	**99.25278**	**114.2563**
**LcP3**	38596873	41738311	37591506	**100.5602**	**108.7449**	**97.94086**
**CtlP1**	39071437	47628208	42718672	**101.7967**	**124.0905**	**111.2992**
**CtlP2**	42613112	34929593	37602818	**111.0241**	**91.00552**	**97.97033**
**CtlP3**	39434979	37903772	38160869	**102.7438**	**98.75444**	**99.42428**
**Mock:** 38381841						

**Peptide concentration: 25 μg mL^{-1}**						
**Sample**	**Cell viability**			**Percentage of cell viability**		
**LrP1**	39632899	38973687	28254288	**99.4643**	**97.80995**	**70.9081**
**LrP2**	38679411	31348368	34822648	**97.0714**	**78.67313**	**87.39233**
**LrP3**	38078693	44468358	33660753	**95.5638**	**111.5996**	**84.47639**
**LcP1**	31867723	34582361	41973477	**79.9765**	**86.78930**	**105.3383**
**LcP2**	41146158	39052769	35938920	**103.2620**	**98.00841**	**90.19377**
**LcP3**	33136835	39395638	43409878	**83.16154**	**98.86889**	**108.9431**
**CtlP1**	42730838	38607934	40469730	**107.2390**	**96.89204**	**101.5644**
**CtlP2**	38607934	39486199	44389277	**96.89204**	**99.09617**	**111.4011**
**CtlP3**	43161758	42205689	36079851	**108.3205**	**105.9211**	**90.54746**
**Mock:** 39846342						

**Peptide concentration: 12.5 μg mL^{-1}**						
**Sample**	**Cell viability**			**Percentage of cell viability**		
**LrP1**	39637461	38899411	36344316	99.07969	97.23482	90.84799
**LrP2**	39266154	39395638	33990007	98.15155	98.47522	84.96304
**LrP3**	36187571	40122656	39559713	90.4561	100.2925	98.88535
**LcP1**	38360042	41610004	41146158	95.88659	104.0103	102.8509
**LcP2**	38360042	41681482	39947003	95.88659	104.1890	99.85343
**LcP3**	38135394	42571914	45855333	95.32505	106.4147	114.6221

**CtIP1**	40641251	46517644	44468358	101.5888	116.2777	111.1552
**CtIP2**	38255867	42718671	34170983	95.6261	106.7816	85.41542
**CtIP3**	39440891	43041933	37735813	98.58833	107.5896	94.32624
**Mock:** 40005636						
